# Advancing Treatment of Bone Metastases through Novel Translational Approaches Targeting the Bone Microenvironment

**DOI:** 10.3390/cancers14030757

**Published:** 2022-02-01

**Authors:** Nan Sethakorn, Erika Heninger, Cristina Sánchez-de-Diego, Adeline B. Ding, Ravi Chandra Yada, Sheena C. Kerr, David Kosoff, David J. Beebe, Joshua M. Lang

**Affiliations:** 1 University of Wisconsin Carbone Cancer Center, University of Wisconsin-Madison, Madison, WI 53705, USA; nsethako@medicine.wisc.edu (N.S.); eheninger@wisc.edu (E.H.); sanchezdedie@wisc.edu (C.S.-d.-D.); ading3@wisc.edu (A.B.D.); skerr2@wisc.edu (S.C.K.); dkosoff1@medicine.wisc.edu (D.K.); djbeebe@wisc.edu (D.J.B.); 2 Division of Hematology/Oncology, University of Wisconsin-Madison, 1111 Highland Ave., Madison, WI 53705, USA; 3 Department of Medicine, University of Wisconsin-Madison, Madison, WI 53705, USA; 4 Department of Pathology and Laboratory Medicine, University of Wisconsin-Madison, Madison, WI 53705, USA; ryada@wisc.edu; 5 Department of Biomedical Engineering, University of Wisconsin-Madison, Madison, WI 53705, USA; 6 Wisconsin Institutes for Medical Research, 1111 Highland Ave., Madison, WI 53705, USA

**Keywords:** bone metastases, tumor microenvironment, 3-D tumor-on-chip models, clinical trials targeting bone metastases

## Abstract

**Simple Summary:**

Solid tumors such as prostate, breast, and lung cancers frequently spread to bone, causing severe pain, disability, and cancer-related deaths. The multiple types of non-cancerous cells in the bone interact with tumor cells to reduce the response to cancer therapies and promote further cancer growth. Studies of cellular interactions in this environment are needed in order to discover new therapies to treat and inhibit bone metastases. This review summarizes the current state of approaches used to study bone metastases, important pathways that could potentially be therapeutically targeted, and the status of clinical investigations of new drugs to treat bone metastases.

**Abstract:**

Bone metastases represent a lethal condition that frequently occurs in solid tumors such as prostate, breast, lung, and renal cell carcinomas, and increase the risk of skeletal-related events (SREs) including pain, pathologic fractures, and spinal cord compression. This unique metastatic niche consists of a multicellular complex that cancer cells co-opt to engender bone remodeling, immune suppression, and stromal-mediated therapeutic resistance. This review comprehensively discusses clinical challenges of bone metastases, novel preclinical models of the bone and bone marrow microenviroment, and crucial signaling pathways active in bone homeostasis and metastatic niche. These studies establish the context to summarize the current state of investigational agents targeting BM, and approaches to improve BM-targeting therapies. Finally, we discuss opportunities to advance research in bone and bone marrow microenvironments by increasing complexity of humanized preclinical models and fostering interdisciplinary collaborations to translational research in this challenging metastatic niche.

## 1. Clinical Burden of Bone Metastases

Within solid tumors, prostate and breast carcinomas have greater tropism for bone, involved in up to 80–90% of patients with metastatic disease [1]. Breast (BC) and prostate cancers (PC) represent the most common solid tumors in female and male individuals, respectively, in the US, followed by lung cancers (LC) [2], and these tumor types commonly metastasize to bone [1]. As such, bone metastases (BM) affect a significant proportion of patients with cancer and contribute to lethality. BM arise most commonly in highly vascularized bone containing red bone marrow in adults and cancellous bone (e.g., bones forming the axial skeleton [3]. Bone lesions can generate skeletal instability, increasing risks of pathologic fractures and spinal cord compression, causing pain and paralysis if not treated expeditiously. There are tumor-specific differences in BM subtypes, as PC favors osteoblastic and sclerotic lesions promoting abnormal bone deposition and sclerosis, whereas many other tumors skew towards osteolytic lesions with increased osteoclastic activity [1]. Table 1 summarizes the frequency and quality of BM, and the incidence of skeletal-related events (SREs) in different solid tumors with advanced or metastatic disease. The most devastating SREs include pathologic fractures and spinal cord compression. The frequency of these is noted specifically in Table 1. Other SREs include pain, need for palliative procedures such as radiotherapy, ablation, or surgery, and malignant hypercalcemia. 

The heterogeneity reported in BM percentages in Table 1 is due to several reasons including different sample sizes and populations, research method (analysis of SEER databases vs. single-institution analysis), improvements in imaging modalities including PET/CT and bone scans, clinically detected BM compared to subclinical disease found in autopsy studies, and overall advances in cancer therapies and survival (thereby allowing more time to develop atypical metastases). The frequency of BMs can vary drastically depending on specific tumor subtypes, and whether studies examined patients with de novo BM or those that emerged throughout multiple therapies. Within a solid tumor type, molecular tumor alterations may further determine the type of bone lesions. For example, non-small cell lung cancer (NSCLC) with ALK (anaplastic lymphoma kinase) rearrangements showed a higher frequency of sclerotic bone lesions compared to NSCLC with EGFR-activating mutations, or NSCLC lacking either of these targetable alterations [4]. A retrospective analysis of patients with bone only metastases in BC showed that the dominant subtype was hormone-receptor-positive and HER2-negative [5]. 

Hematologic malignancies including leukemia, lymphoma, and multiple myeloma (MM) frequently involve the skeletal system. Leukemias can cause bone pain, fractures, and osseous radiographic abnormalities [6,7,8,9,10]. However, clinical manifestations are frequently related to profound cytopenias and coagulation disorders secondary to malignancy, as well as treatment-induced ablation of the normal hematopoietic stem cell niche [11,12]. Hematologic malignancies consist of heterogeneous subtypes based on the cell of origin and pediatric vs. adult manifestations, but have unique cancer-stromal interactions within the specialized hematopoietic bone marrow niche (comprehensively reviewed by Mendez-Ferrer, et al.) [13]. Skeletal complications in lymphoma relate to long-term osteoporosis and insufficiency fractures secondary to premature bone loss from lymphoma therapies such as glucocorticoids and chemotherapy [14], although osteolytic lesions have been reported [9]. Myeloma bone disease shares similar clinical complications seen in solid tumor BM (described in Table 1) and has been reviewed elsewhere [15].

In most cases, the presence of bone involvement confers a poor prognosis [1,5,16,17,18,19,20,21,22,23,24]. It is possible that this reflects consequences of higher disease burdens; however, specific bone involvement could promote biological pathways that increase tumor aggressiveness. The associated morbidity can frequently preclude administration of additional life-prolonging therapies. Patients with multiple BM had worse prognoses that those with limited BM in a retrospective analysis of patients with bone-only metastatic BC [25]. In some instances, such as hormone receptor-positive BC with limited sites of bone-only metastases and no visceral organ metastases, this represents a patient population with improved prognosis [26]. 

**Table 1:** Characteristics of bone metastases in diverse solid tumors. BM = bone metastases, SRE = skeletal related event, NSCLC = non-small cell lung cancer, SCLC = small cell lung cancer, HCC hepatocellular carcinoma, NR not reported. * indicates frequency including all stages of that malignancy if frequency in advanced/metastatic disease was not specifically reported. ** indicates inclusion of data reported from autopsy series. SRE frequency was reported as the number of patients who experienced an SRE as a percentage of patients with clinically detected bone metastases.

	Frequency of BM in Advanced Disease	Predominant Type	Frequency of SRE
Prostate [17,18,23,27,28]	80–90%	Blastic	22% (pathologic fracture) 31–48%
Breast [29,30,31,32]	65–80%	Lytic	39% (pathologic fracture) 40–47%
NSCLC [20,33,34,35]	30–60%	Lytic	38–63% 20% (pathologic fracture) 10% cord compression
SCLC [36,37]	50–66%	Lytic	8–34%
Renal cell [38,39,40]	20–68%	Lytic	70–85% 28% (spinal cord/nerve root compression)
Urothelial ** [41]	32–47%	Lytic	7%
Melanoma [42,43,44]	17–52%	Lytic	47–58%
Thyroid [45,46,47,48,49]	4 *–50%	Lytic	32–78%
HCC [50,51,52,53]	4.5–38%	Lytic	56%
Biliary tract [54,55,56,57]	2–35%	Lytic	41% 16% (pathologic fracture) 8% (cord compression)
Gastric ** [58,59,60]	4–45%	Lytic	31–75%
Esophageal ** [61,62,63]	15–24%	Lytic	91%
Colorectal [64,65,66]	3–24%	Lytic	62–68% 8–10% (pathologic fracture) 6–9% (cord compression)
Pancreatic [67,68,69]	2–12%	Blastic	32–57% 6% (pathologic fracture) 3% (cord compression)
Squamous cell carcinoma of the head and neck [70,71,72,73]	1–16%	Lytic	9–31% 2–12% (pathologic fracture) 6–7% (cord compression)
Endometrial ** [74,75,76]	1 *–25%	Lytic	NR
Ovarian ** [77,78,79]	1 *–15%	NR	NR
Soft Tissue Sarcomas [80,81]	9–11%	Lytic	40% 22–31% (pathologic fracture) 13% (cord compression)
Multiple Myeloma [82,83,84,85,86]	80–90%	Lytic	22–60% 14–34% (pathologic fracture) 4.7–7.8% (cord compression)

In contrast, several studies reported poor therapeutic response in patients with BM. In patients with NSCLC treated with the checkpoint inhibitor nivolumab, those with BM had worse survival and less chance of responding to treatment [24]. Similarly, ipilimumab treatment was less effective in patients with metastatic castrate-resistant PC with BM compared to those without [87,88]. Patients with EGFR-driven lung adenocarcinoma experienced limited sites of progression while on therapy with an effective EGFR inhibitor. These sites were all in bone, and effectively treated with high-dose ablative radiotherapy. Patients continued systemic treatment with the same EGFR inhibitor with durable disease control [89]. BM was associated with worse survival in patients with melanoma receiving either targeted or immunotherapy, independently of the number of sites and other clinical parameters [90]. Bone stroma confers resistance to systemic therapies, either through secretion of paracrine factors or direct cell-cell contacts [91]. Altogether these data indicate that the bone and bone marrow niche is a unique microenvironment that permits tumor growth and treatment resistance. 

Bone and bone marrow represent distinct niches compared to other sites in patients with metastatic solid tumors; however, there is limited clinical data on bone responses. This is due, in part, to challenges in obtaining and processing BM from patients and difficulty assessing radiographic responses in bone. A sclerotic appearance may indicate a favorable response to targeted therapy, immunotherapy, or chemotherapy in NSCLC [92,93,94]. Jiao, et al., compared transcriptional profiles of primary prostate versus bone marrow biopsies in patients with metastatic castrate-resistant PC, pre and post treatment with ipilimumab. The T cell populations differed between sites, with a greater degree of immunosuppressive polarization within bone marrow. This was recapitulated during inoculation of murine PC cells either in subcutaneous or intraosseous pockets of syngeneic mice [87]. Exploratory analysis of bone marrow aspirates in a single-arm phase I clinical trial of patients with metastatic PC receiving a dual checkpoint inhibitor with tremelimumab and durvalumab demonstrated upregulation of myeloid and neutrophil signatures after immunotherapy [95]. TGF-beta was highly enriched in both patient and murine bone marrow supernatant with BM. Consistent with these findings, there is a higher frequency of regulatory T cells (T_regs_) in the bone marrow aspirates of patients with bone metastatic PC, but not from age-matched donors without cancer [96].

A major challenge to assessing efficacy is the endpoint used in clinical trials. BM are typically considered non-measurable, which limits the ability to assess tumor responses by standard CT imaging. This important topic is discussed by Hussain et al. [1]. Briefly, endpoints include pain response, patient quality of life, bone density, time to disease progression and/or SRE, and overall survival. In cancers with a well-defined tumor marker, such as PSA in PC, a reduction in PSA is a common surrogate endpoint. Advances in nuclear medicine imaging modalities could also provide an additional approach to measure tumor response in bone. Improving technology in detection of circulating biomarkers including circulating tumor cells, cell-free tumor DNA, or tumor-derived exosomes are emerging as novel correlative markers of disease response.

Currently there are limited treatments for specific management of BM. Radiopharmaceuticals such as radium-223 deposit only in bone and are approved in PC with bone-only metastasis; however the effect on prolonging survival is modest [97]. Agents that prevent bone resorption include zoledronic acid, a bisphosphonate that inhibits the production of RANK-ligand (RANKL) and stimulates synthesis of osteoprotegerin (OPG), and denosumab, an antibody that inhibits the osteoclast-promoter RANKL [98,99,100,101]. These agents have minimal effect on overall survival, but prevent and delay SREs. Focused external beam radiation therapy is effective in palliating pain and can prevent further progression at the sites of irradiation. In some cases, surgical intervention is required to decompress the spinal cord or stabilize pathologic fractures. Newer approaches involve percutaneous ablation and vertebroplasty as a less invasive interventional method to reinforce bone at risk for pathologic fractures and palliate pain [102]. Therefore, advances in systemic treatment of BM are acutely needed. 

## 2. Complex Multicellular Composition of Bone and Bone Marrow Metastatic Niches

**Figure 1:**
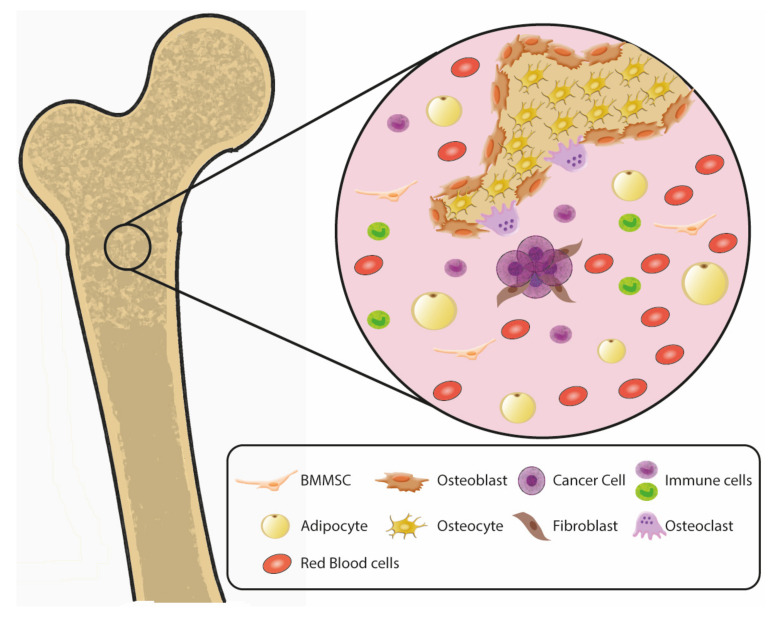
Multicellular composition of the bone microenvironment. The periosteal niche contains cells that regulate bone formation, deposition, and breakdown. These cell types release multiple growth factors and cytokines. The bone microenvironment is highly vascularized and immune rich, with immunosuppressive tendencies to protect the hematopoietic stem cell components. These factors generate a highly tumor-permissive environment to favor cancer cell metastasis. BMMSC = bone marrow mesenchymal stem cell.

**Figure 2:**
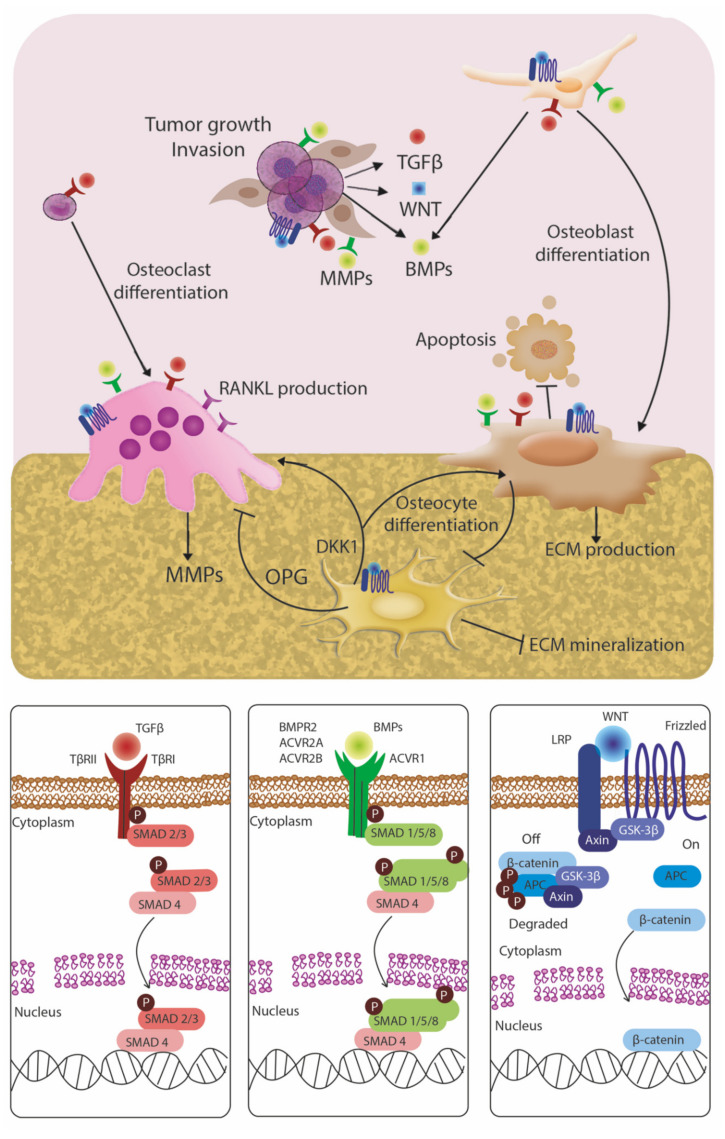
Paracrine signaling pathways active in the bone niche. Under nonmalignant conditions, these promote normal bone homeostasis, balancing bone deposition/mineralization and breakdown. TGF-beta, BMP, and Wnt signaling pathways are highlighted as key mediators of paracrine signaling between bone stromal cells and tumor cells. Multiple cancer cell types co-opt these pathways to promote metastasis to bone, develop treatment resistance, and enhance cancer cell survival. Multiple approaches to inhibiting these pathways include small molecule inhibitors, antibodies, and other types of antagonists.

Bone is highly vascular and multicellular with extensive cell-cell and paracrine interactions, as depicted in Figure 1 and Figure 2. Bone marrow-derived mesenchymal stem cells (MSCs) are multipotent cells that can differentiate into bone-forming osteoblasts, which become osteocytes as they are encased in mineralized matrix proteins secreted by osteoblasts. MSCs also have chondrogenic, fibrogenic, adipogenic, myogenic, and tenogenic abilities. MSCs induced Akt-dependent invasion in PC cells [103], and increased chemoresistance mediated by integrin signaling [104]. Cancer cells can condition MSCs, since a co-culture of PC cells induced osteogenic markers in MSCs [104], and tumor-induced bone secreted multiple targetable factors [105,106,107]. Osteoblast-tumor interactions promote BM, and cancer cells frequently disrupt bone homeostasis to promote lytic or sclerotic lesions [108,109]. 

No longer considered passive placeholders in bone, osteocytes regulate bone remodeling and mineralization by secreting osteoclast activators (RANKL), osteoclast inhibitors (e.g., OPG) and bone formation inhibitor sclerostin (Sost) [110,111]. Initially, osteocytes enhance bone mineralization and reduce osteoclast recruitment and activity. As a response to tissue damage during metastasis, osteocytes release extracellular ATP (eATP) [112], which inhibits the growth of several cancer cell types including pancreatic, colon, prostate, breast, liver, ovarian, colorectal, esophageal, melanoma, and leukemia [113]. However, tumor cells can modify osteocyte activity, inducing osteoclast activators (e.g., RANKL) and Wnt inhibitors (e.g., Sost and Dkk1) that hamper osteoblast differentiation. MM cells stimulate Sost and RANKL in osteocytes, as well as pre-osteocyte autophagic death, leading to bone loss and MM growth [114,115,116]. Through direct cell-cell contacts, paracrine factors, and tumor-generated physical forces, tumor cells stimulate secretion of growth factors, (e.g., growth-derived factor 15 (GDF15)), chemokines (e.g., chemokine (C-C motif) ligand 5 (CCL5)) and metalloproteases, that promote proliferation, migration, and invasion of several cancer types including prostate, breast and lung [117,118].

The bone marrow is considered a secondary lymphoid organ that provides a niche for vast networks of innate and adaptive immune cells and regulates immune homeostasis. While bone marrow immune cells exert robust antitumor activity, cancer cells can subvert the functional integrity of bone marrow immunity [119,120]. It has been hypothesized that this subversion could be promoted by the immune suppressive nature of this milieu and immune-osseous interactions that regulate bone homeostasis. To nurture a hematopoietic stem-cell (HSC) niche and shield it from environmental stressors, bone marrow provides a hypo-immunogenic milieu enriched in regulatory T cells, myeloid-derived suppressor cells (MDSCs) and immune suppressive factors. This immune-privileged site maintains a distinct immune composition including an increased T cell to B cell ratio (5:1) and decreased CD4:CD8 ratio (1:2) [121,122,123]. Most lymphocytes exhibit an antigen-experienced activated memory phenotype, while the frequency of naïve T cells is largely reduced compared to spleen or lymph nodes. Bone marrow preferentially retains T_reg_ cells that can make up to ~25–30% of the CD4 subset, which can be further enriched in metastatic cancer [96]. Regulatory T cells have profound effects at suppressing anti-tumor CD8 T cell and NK cell responses and reduce IFN-gamma production. Additionally, T_reg_ cells can promote the ‘vicious cycle’ by suppressing osteoclasts and promoting osteoblastic PC dissemination. Th17 cells regulate tissue remodeling and bone reabsorption and may support both an antitumor activity or promote metastatic pathogenesis. Preferential expansion of Th17 polarization was identified in bone marrow of patients with PC with BM undergoing anti-CTLA4 immune therapy (NCT02703623 and NCT02985957) in contrast with an enhanced Th1 signature in patients with soft-tissue metastasis [87]. 

Unconventional T cells, such as gamma-delta T cells and NKT cells in bone marrow, have significant roles in tumor immune surveillance and promising therapeutic potential in treatment of metastatic PC. Gamma-delta T cells produce IFN-gamma, promote antitumor T cell activity, directly suppress osteoclasts, and have been associated positive clinical outcomes in PC [124]. NKT cells exhibit potent antitumor activity and are greatly reduced in PC tissue [125] and in PC bone marrow [126]. NKT cells rapidly diminish at the periphery after antigen encounter and get replenished in bone marrow. Therefore, bone marrow also plays a central role in NKT homeostasis during antitumor responses. Changes in homeostasis and function within the complex T cell networks may play a robust, multi-faceted role in dissemination of tumor cells to the bone marrow. For example, disruption of the CCL20-CCR6 axis prolonged survival and enhanced T cell reactivity in a syngeneic mouse model of metastatic PC [127]. Further research is needed to explore potentially actionable mechanisms to revert immune suppression and prevent invasion. 

Macrophages are another cell population that are highly important in development of BM. Preclinical studies demonstrated that macrophage depletion decreased formation of skeletal metastases in vivo, indicating that macrophages support establishment of bone metastatic foci as well a progression of these lesions [128,129]. While mechanisms by which macrophages promote BM continue to be under investigation, available data suggest that these pathways are multifaceted due to wide-ranging functions of macrophages within bone [130]. Macrophages are an extremely diverse cell population with the ability to adapt their phenotype to the needs of each microenvironment [131]. Within the bone microenvironment there are multiple populations of macrophage-derived cells, each with a unique set of phenotypic characteristics and physiologic functions. 

Osteoclasts are among the most well described population of macrophage-derived cells within the bone marrow. Their primary function is bone resorption, which is a critical component of the bone remodeling process that continually occurs to maintain bone structure [132]. In addition to osteoclasts, osteal macrophages or “osteomacs” are another resident macrophage-derived population involved in bone remodeling. These cells constitute approximately 17% of the bone marrow cells and they differ from osteoclasts by the expression of CD68 [133]. They are primarily located around osteoblasts and support bone homeostasis by phagocytosing aging osteoblasts, and secrete cytokines stimulating the growth of newer osteoblasts [130]. Bone marrow derived macrophages (BMDMs) and monocyte derived macrophages (MDMs) can also be recruited into the bone microenvironment. These cells can support a range of functions in the bone marrow, ranging from bone remodeling and cell growth to immune regulation and angiogenesis [134,135].

During development of BM, tumor-derived factors alter functions of various macrophage populations within the bone microenvironment to support tumor cell survival and proliferation. This process begins prior to the arrival of tumor cells when cytokines secreted by the primary tumor, such as VEGF, TNF-alpha, and TGF-beta, recruit immunosuppressive myeloid cells to the bone [136]. Primary tumors also secrete exosomes, which contain an array of factors (microRNA, proteins, among others) that increase macrophage-mediated efferocytosis and osteoclastogenesis in anticipation of tumor cell arrival [137,138]. Once present in the bone, tumor cells produce TGF-beta and parathyroid hormone-related peptide (PTHrP) to facilitate migration by inducing macrophage mediated remodeling of the surrounding ECM [139]. Tumor cells within the bone are also able to recruit CD68+ cells from the blood and bone marrow into the TME [140]. These tumor-associated macrophages (TAMs) support tumor growth and survival through the same TAM-mediated pathways that are active in the primary tumor and other metastatic sites (i.e., secretion of growth factors, angiogenesis, immune suppression) [135]. Additionally, efferocytosis of apoptotic cancer cells by macrophage populations leads to production of an array of factors, including CXCL5, IL-6, IL-8, CCL4, and CCL5, which promote tumor growth and survival [141,142].

Adipocytes account for up to 60% of cells in bone marrow, but despite their abundance their role in BM has not been well characterized. Recent data suggest that adipocytes regulate tumor growth in BC and promote melanoma metastasis [143,144]. Bone marrow adipocytes (BMAs) secrete adipokines (e.g., CXCL12, CXCL10 and CX3CL1,) that increase vascular permeability and act as chemoattractants for tumor cells [143,144,145]. The release of proinflammatory cytokines (e.g., IL-1beta, IL-6, TNF-alpha,) suppresses myeloid-derived cells, and therefore inhibits both the innate and adaptive immune response [146]. Once metastasis is established in the bone marrow, BMAs release free fatty acids to provide energy and support the growth of tumor cells. Treatment with ND-646, an inhibitor of acetyl-CoA synthesis, reduced cancer cell lipid droplets and decreased tumor cell proliferation in preclinical models of NSCLC [147]. BMAs perturb bone turnover, activate osteoclast differentiation through adipocyte-derived RANKL, CXCL1, and CXCL2, and suppress osteoblast differentiation by inhibiting bone morphogenic protein (BMP) signaling [148,149,150]. 

## 3. Models of Bone Metastasis

A major challenge in tumor microenvironment modeling remains balancing tissue complexity and scalability, particularly relevant considering the complex cellular and matrix components of bone and bone marrow [151]. Multicellular environments with three-dimensional (3-D) properties require higher levels of technical expertise and often are associated with increased labor intensity to maintain in vivo mouse models or obtain and culture primary human tissue. Development of novel systemic therapies to prevent or inhibit BM, however, requires a detailed understanding of stromal-tumor interactions. The bone microenvironment itself is highly complex and is comprised of multiple niches, including the osteogenic niche, hematopoietic stem cell niche, and perivascular niche. This section highlights commonly used in vivo and ex vivo models and discusses strategies to improve modeling of the bone microenvironment.

### 3.1. In Vivo Models

Intracardiac injection of tumor cells reliably generates BM compared to other inoculation routes, although pretreatment of mice with melanoma-derived exosomes generated BM even with subcutaneous implantation of melanoma cells [152]. Shiozawa, et al., used an in vivo model in which osseous vossicles (vertebral bodies from 4–7 days old wild type or mice expressing an inducible collagen transgene) were implanted subcutaneously into SCID mice, facilitating assessment of metastatic tumor cells to bone and allowing bone specific ablation of collagen to target the endosteal niche [153]. Intra-iliac injection also results in BM, and this technique has been combined with bone-in-culture ex vivo arrays using fragmented murine BM to interrogate multiple therapies [109]. Live animal imaging through mouse calvarium enabled visualization of human BC cell homing to specific bone niches after intracardiac injection in immunocompromised mice [154]. Intraosseous injection can model local tumor-stromal interactions but does not address the early stages of metastasis. A detailed description of murine BM protocols was summarized by Park, et al. [155]. Mouse models have led to several important discoveries in signaling pathways mediating BM, are valuable in preclinical drug development but require a high degree of technical complexity and higher costs, and should be interpreted with an understanding of the differences between mouse and human hematopoietic and lymphoid lineages [156]. 

The human immune and stromal components within bone and bone marrow were not captured in previous in vivo models due to the use of immunocompromised or syngeneic mice. Humanized mice with reconstituted hematopoietic compartments have enabled significant advances in modeling preclinical immuno-oncology therapies. However other regions of the bone remain of mouse origin [157]. Several groups have incorporated specific human microenvironments in immunocompromised mice. For instance, McGovern, et al., incorporated human cancer-associated fibroblasts and lymphatic and blood endothelial cells in an orthotopic model of PC, combined with subcutaneous implantation of humanized tissue-engineered bone constructs (hTEBC) [158]. Using this same model, a xenograft of a PC cell line responded to zoledronic acid but not denosumab (a human-specific RANKL antibody) [159]. This suggests that this model may lack human osteoclasts, the target of RANKL inhibitor denosumab, which was as efficacious as, or better than, zoledronic acid in clinical trials [160,161]. Chen, et al., injected human MSCs and endothelial cells embedded in Matrigel into immunocompromised mice, and subsequently introduced human cord blood cells intravenously. This method simulated extramedullary bone with human stromal cells engrafted with human hematopoietic cells [162]. Lee, et al., advanced a similar approach with genetic engineering of human stromal cells to assess how distinct cytokines modulate HSC function and recruitment [163]. Additional models with implantation of humanized bone marrow niches in the study of bone marrow engraftment, hematopoiesis, and leukemia have been reviewed by Abarrategi, et al. [164].

### 3.2. Bioengineered Microfluidic Models

Microfluidic three-dimensional (3-D) tissue chip platforms have emerged as a promising method to bridge the gap between two-dimensional (2-D) coculture and in vivo methods and improve modeling of metastatic niches [165]. These platforms can accommodate the culture of multiple cell types in a microenvironment, replicate the tumor architecture, generate physiologically relevant nutrient/chemokine gradients, and have tunable components that can control mechanical cues or stresses that can directly influence cell behavior and function [166]. Furthermore, they enable low-input cell culture, which increases the analytical endpoints that can be obtained from a limited resource such as primary human cells. High optical imaging capacity using confocal microscopy enables detailed 3-D visualization of cancer cell extravasation and intravasation. It has been well described that the 3-D environment afforded by these platforms helps to better recapitulate cell polarization, and crosstalk between metastatic cells and the niche [167]. The increased complexity of ECM organization in microfluidic models accentuates the migration and proliferation advantages metastatic cells have in vivo compared to their 2-D counterparts [168,169]. The interaction of tumor and endothelial cells is an often understated aspect of modeling the metastatic niche. The addition of microvasculature into 3-D BM microfluidic models further captures the dynamics of cell recruitment and signaling in the microenvironment, which is particularly relevant as malignant angiogenesis is a hallmark of cancer cell growth, and led to development of anti-angiogenic agents as part of cancer therapies in multiple solid tumor types [170]. Existing 2-D coculture systems, 3-D static and dynamic in vitro platforms fail to account for this integral interaction and signaling. Microfluidic systems are uniquely positioned to incorporate 3-D tumor spheroid cultures into an ECM matrix with embedded stromal cells alongside microvasculature. The effect of secreted factors by the growing cancer on the function of the surrounding bone niche and endothelial vessels can be better explored in advanced microfluidic devices. Additionally, microfluidic models enable passive pumping or active pumping methods within lumens to investigate both the effects of increased shear stress in the microenvironment, as well as the dynamics of cell extravasation in the context of a growing metastatic bone niche [171,172]. A comprehensive and well-crafted review by Laranga, et al., highlights many of the pros and cons of different BM methods, including the significant advantages microfluidic devices have in generating model systems for investigating a plethora of both biologically and clinically relevant questions [173]. Importantly, the ease of use, customizability, and 3-D coculture capabilities of microfluidics lends to its increased adoption compared to more expensive, and less reproducible scaffold based [174] or bioreactor systems [175] commonly employed for studying metastatic microenvironments.

Bioengineered bone is actively being pursued in both cancer research and primary bone disease such as osteoporosis and tissue regeneration. One group used primary human osteocytes cultured in 3-D with microbeads as a physical support, coupled with a passive pumping system to continuously replenish nutrients and excrete waste. They demonstrated bone mineralization of the system, and that coculture with PC cells altered Wnt and fibroblast growth factor signaling that was not recapitulated in 2-D coculture [176]. Bioreactor-cultured human bone fragments containing osteoblasts, osteocytes, osteoclasts, adipocytes, and hematopoietic progenitors were cocultured with BC cells, which upregulated osteoclastic cytokines consistent with lytic BC bone lesions [177]. Sieh, et al., used medical grade polycaprolactone-tricalcium phosphate scaffolds (used clinically as cranial implants) embedded with human osteoblasts as a bone construct to show that this structure induced expression of androgen-responsive genes in a PC cell line even in the absence of an androgen mimic [178]. A bioengineered bone-mimetic environment demonstrated stroma-mediated chemoresistance and recapitulated localization of radium-223 [179]. Another group developed a platform called human osteoblast-derived tissue-engineered construct (hOTEC) that recapitulated encased osteocytes within mineralized tissue and was cocultured with a PC PDX [180]. Other microscale chips incorporated osteoblastic cells derived from MSCs and showed that these cells could mineralize the surrounding 3-D matrix, inducing BC cell invasion [181,182]. Coculture of MSCs and endothelial cells formed a vascularized, mineralized structure enabling tracking of PC motility [183]. 

Structurally complex environments have been generated by incorporating luminal structures lined by endothelial cells to mimic vasculature or epithelial cells to mimic ductal structures [184,185]. Culturing cells in a physiologically relevant geometry, as found in these models, can have a profound impact on cell function and production of secreted factors [186]. Similar methods have been used to visualize fibrosarcoma and BC cell extravasation across a microfluidic endothelial barrier and extracellular matrix [187]. BC cells have also been seeded within an endothelial-lined lumen structure and observed to intravasate through this structure towards osteoblasts encapsulated in a 3-D matrix [181]. These studies represent an important advancement in recapitulating endothelial-stroma-cancer cell crosstalk; however, there are some limitations. First, most of these studies focus on specific stromal cells—fibroblasts, osteoblasts, osteoclasts, or adipocytes. As discussed, the bone niche is comprised of multiple cell types, and thus its complexity is challenging to capture ex vivo. Parikh, et al., described an ex vivo mixed, multicellular system expanded from bone marrow mononuclear cells that spontaneously generate osteoblasts, osteoclasts, adipocytes, and fibroblasts in the same culture [188]. Therefore, embedding these mixed cultures within a microfluidic bone tissue chip could bypass this limitation, and incorporating luminal structures can recreate the bone endosteal/perivascular niches. 

Most of the models described above enrich for stromal cells such as osteoblasts and stromal progenitors. Additional methods are required to enrich for the hematopoietic and stem cell compartments. Torisawa, et al. [189] generated a PDMS device engineered to create a physical bone mineralized structure derived from syngeneic implanted subcutaneously in mice. Over several weeks, hematopoietic cells infiltrated, and its cellular content resembled murine bone marrow histologically and immunophenotypically. This structure could then be retrieved and cultured in vitro within a microfluidic device up to seven days, with appropriate responses to radiation and granulocyte colony-stimulating factor. Recently, Glaser, et al. [190] developed a multichamber 3-D organ-on-chip model that demonstrated coculture of an osteoblast cell line with primary human stromal cells, and human hematopoietic stem progenitor cells. This chip recapitulated bone niches that incorporated vascular networks, maintained hematopoietic progenitor cell function, responses to chemotherapy and granulocyte colony-stimulating factor, neutrophil egress, and BC cell line invasion into the bone niches. Increasing the cellular complexity to incorporate stromal, vascular, progenitor stem, immune, and tumor cells will enhance the ability to replicate a human microenvironment. Platforms incorporating this complexity will enable ex vivo analysis of factors mediating response or resistance to tumor immunotherapies in humanized models.

## 4. Signaling Pathways Mediating Bone Metastasis and the Status of Clinical Trials of Biologic Agents Targeting Bone Metastases

Bone metastasis is a complex, multistep process, and a recent review by Zhang, et al., provides a global summary of the bone niche in solid tumors [191]. In this section we highlight prominent signaling hubs involved in disseminated tumor cells to provide context for rational clinical targeting of these pathways and discuss the current status of clinical trials targeting these pathways. Importantly, many of the signaling pathways crosstalk with each other, providing a potential avenue to target “bone metastatic signaling hubs”, which may be more effective than ablating individual components. It is hypothesized that targeting the pathways that promote BM will reduce metastatic burden and prevent the environment-mediated treatment resistance from the tumor-permissive bone stroma. 

As discussed above, antiresorption therapies have primarily been shown to prevent or delay SREs in patients with bone metastatic cancers. A meta-analysis of patients with localized hormone receptor positive BC demonstrated that zoledronic acid given in the adjuvant setting after mastectomy modestly reduced the risk of BM [192]. Table 2 summarizes recent trials targeting solid tumor BM that are complete or in progress. Most of these trials were in early phases and thus outcomes focused primarily on safety; however, early data suggest only modest, limited antitumor activity. Given that the mechanisms in BM cover diverse pathways, it is likely that combination therapies targeting both the tumor and stroma will be more effective than single agents. Additionally, many of these agents are tested in patients who have been heavily pretreated and already have multiple metastases, raising the hypothesis that targeting tumor-stromal interactions at this later stage may not confer meaningful clinical benefit. Therefore, trials that incorporate safety and efficacy of bone targeting agents earlier in the disease course may be more effective, and understanding the effects of systemic therapies on bone deposition/resorption must be considered. For example, combining radium-223 with abiraterone (an inhibitor of androgen synthesis) increases the risk of fractures in PC and underscores the caution necessary in this setting [193]. In contrast, a phase I trial evaluated the addition of radium-223 to standard FDA-approved anti-angiogenic agents in renal cell carcinoma [194]. Combination therapy decreased bone turnover markers and showed early promising activity, leading to a larger randomized phase II clinical trial of radium-223 and cabozantinib in this cancer (NCT04071223). Lastly, these trials included a broad range of patients without selection of particular biomarkers that could predict response to targeted therapies. Thus, evaluation of multiple exploratory biomarkers in these early trials may guide selection of patients for future investigations.

**Table 2:** Status of select clinical trials targeting bone metastasis pathways. ORR overall response rate, DLT dose limiting toxicity, MTD maximum tolerated dose, CR complete response, PR partial response, SD stable disease, PFS progression free survival, OS overall survival, CBR clinical benefit rate (PR rate+ rate of SD at least 6 months), BC breast cancer, mCRPC metastatic castrate resistant prostate cancer, ZA zoledronic acid, SCLC small cell lung cancer, NSCLC non-small cell lung cancer, RCC renal cell carcinoma, MM multiple myeloma, MMP matrix metalloproteinase, ET_A_ endothelin-A.

	Drug	Drug Class	Molecular Target	Phase	Trial Number	Disease Type	Results
Chemokine ligand or receptor	Balixafortide + eribulin	Targeted therapy + non-taxane mitotic inhibitor	Balixafortide—selective CXCR4 antagonist	I	NCT01837095	HER2-negative BC	ORR 30%, MTD not reached [195]
	Carlumab	Monoclonal antibody	CCL2	I/II	NCT00992186	mCRPC, advanced solid tumors	9–34% of patients had SD [3] 3 mo, 39% improved pain scores [196,197]
	Reparixin + paclitaxel	Targeted therapy + taxane mitotic inhibitor	Reparixin—CXCR1/2	IB	NCT02370238	HER2-negative BC	30% ORR, no DLTs [198]
	Propagermanium	Targeted therapy	Glycosylphosphatidylinositol-anchored proteins (CCL2 pathways)	I	UMIN000022494	Perioperative BC	No DLTs [199]
	LY2510924 + carboplatin and etoposide	LY2510924—targeted therapy, carboplatin—platinating agent, etoposide—topoisomerase inhibitor	LY2510924—CXCR4 antagonist	II	NCT01439568	SCLC	No difference in PFS, OS, or ORR, no additional toxicity [200,201]
	BMS-986253	Monoclonal antibody	CXCL8 (IL-8)	I	NCT02536469	Advanced solid tumors	No DLTs, 73% of patients had SD with median treatment duration 24 wks (range 4–54). No objective tumor response [202]
TGF-beta	M7824	First-in-class bifunctional checkpoint inhibitor	PD-L1 and TGF-beta	I	NCT02517398	Advanced solid tumors	MTD not reached, 1 CR, 2 PR, 3 SD [203]
	Fresolimumab + focal radiotherapy	Monoclonal antibody	TGF-beta	II	NCT01401062	mBC with at least three distinct metastatic sites	10 mg/kg dose resulted in increased median OS with HR of 2.73 (95%CI 1.02–7.3, *p* = 0.039) compared to 1 mg/kg dose [204]
	Galunisertib vs. placebo, in combination with gemcitabine	Galunisertib—targeted therapy, gemcitabine—antimetabolite	Galunisertib—TGB-beta kinase I inhibitor	Ib/II	NCT01373164	Advanced pancreatic cancer, first-line therapy	Median OS 8.9 and 7.1 mo for G v P, HR = 0.79 (95%CI 0.59–1.09) with posterior probability HR < 1 = 0.93 [205]
Matrix remodeling	BMS-275291	MMP inhibitor	Broad range of MMPs	I	NCT00006229 (CAN-NCIC-BR18); NCT00039104; NCT00040755; NCT00036621	Advanced solid tumors; NSCLC, PC, BC	27% of patients had SD [206,207]
	Marimastat vs. placebo	MMP inhibitor	Broad range of MMPs	III	NCT00003010	mBC	No effect on PFS [208]
	Marimastat + carboplatin and paclitaxel	See above	See above	I	NCT00003011	NSCLC; SCLC	57% PR, 19% SD, tolerable combination therapy in NSCLC [209]. No effect on survival in SCLC [210]
Other	KX2-391	Dual function targeted therapy	Src and tubulin polymerization inhibitor	II	NCT01074138; NCT00658970	Bone mCRPC, chemotherapy-naïve; advanced malignancies	10% PSA response, median PFS 18.6 wks [211]
	Dasatinib	Targeted therapy	Multikinase inhibitor	II	NCT00385580; NCT00918385	mCRPC, chemotherapy-naïve	43% SD rate at 12 wks [212] Significant changes in (18)F-fluoride incorporation in response to Tx [213]
	Dasatinib + ZA	See above	See above	I/II	NCT00566618	HER2-negative bone metastatic BC	23% PR, 36% CBR [214]
	Atrasentan	Targeted therapy	Selective ET_A_ receptor antagonist	II/III	NCT00134056; NCT00181558; NCT00036543; NCT00039429	CRPC, RCC	No effect on in PFS/OS [215]
	BHQ880	Monoclonal antibody	DKK-1	Ib	NCT00741377	Relapsed MM	Increased bone density, tolerable with concurrent MM therapy including ZA [216]

### 4.1. TGF-Beta Family

Transforming growth factor beta (TGF-beta) and Smad signaling induces pleiotropic effects on cancer progression and in bone homeostasis, which are highly dependent upon cellular contexts such as the stage of cell differentiation [217]. In early stages of bone differentiation, TGF-beta promotes osteoblast differentiation and proliferation, blocks osteoblast apoptosis, and enhances extracellular matrix production [217,218,219,220]. In later stages, TGF-beta inhibits osteocyte differentiation and matrix mineralization [221,222]. TGF-beta can also induce differentiation of osteoclast precursors, secretion of osteolytic cytokines, and osteoclastic activity [223]. Therefore, in the appropriate cellular context, TGF-beta blockade may confer dual antitumor and bone protective effects [224]. 

In advanced stages of cancer progression, TGF-beta induces oncogenic, invasive, and immunosuppressive pathways that promote tumor growth. Kang, et al., leveraged in vivo fluorescent and bioluminescence imaging to show increased Smad/TGF-beta pathway activity in bone metastatic BCs induced after intracardiac inoculation [225]. TGF-beta-induced genes include matrix degradation, cell adhesion, and growth factor pathways that contribute to bone destruction and cancer invasion [226,227]. Multiple studies demonstrated that inhibition of TGF-beta through small molecule inhibitors, neutralizing antibodies, or overexpression of inhibitor pathways reduced BM in BC, PC, and melanoma, further highlighting TGF-beta as an attractive target for novel therapies [228,229,230,231,232,233].

Luspatercept, a TGF-beta family blocking fusion protein, was recently FDA-approved for the treatment of anemia secondary to beta-thalassemia, and to reduce red blood cell transfusion requirements in low-risk myelodysplastic syndrome [234,235]. Toxicities included transient bone pain, joint pain, and hypertension. Clinical anticancer activity of this agent remains unclear, though this study demonstrated drug tolerability. Other TGF-beta antagonists are under investigation in advanced solid tumors (Table 2). An immune-modulating agent, tasquinimod, decreased tumor growth, increased antitumor cytokines, and decreased TGF-beta in an intratibial model of bone metastatic PC. However, this agent also increased osteoclastic cytokines, decreased osteogenic genes, and impaired osteoblast mineralization, and therefore must be investigated with caution to avoid unintended bone impairment [236].

### 4.2. BMP Signaling

Bone Morphogenic Proteins (BMPs), members of the TGF-beta superfamily, are essential for differentiation of MSCs into chondroblasts and osteoblasts [237,238]. However, BMPs have a dual role in cancer, with the capacity to inhibit cancer cell growth and enhance cell migration and invasion [239]. BMPs are aberrantly regulated in BM from BC and PC [240], and enhance BC bone invasion [241]. Different BMPs, or the same BMP ligand in different cancer cell types, can promote or inhibit several cancer hallmarks, including cell proliferation, survival, stemness, and migration. In tumor microenvironments, BMPs have a tumor-promoting phenotype, and the use of BMP antagonists reduced PC and BC BM [242,243]. In mouse BC xenografts, knockdown of BMPR1a in BC cell lines suppressed RANKL production, inhibited cancer-induced osteoclastogenesis, and reduced osteolytic metastases [244]. These results suggest that BMPs are potential targets to prevent BM, though potential therapies must be tailored to specific BMP molecules and metastatic cellular contexts.

### 4.3. Wnt Signaling

The Wnt/beta-catenin pathway is crucial in osteogenesis, is associated with invasion and poor prognosis in BC and PC [245,246,247,248], and communicates extensively with the TGF-beta pathway [249,250]. DKK-1, which represses osteoblast-induced Wnt molecules, inhibits osteoblastic differentiation and induces RANKL levels to enhance osteoclastic activity [251]. DKK-1 promotes overall burden of BM, particularly in osteolytic lesions in LC and PC, [252,253] and is highly expressed in BC and PC cell lines that classically produce osteolytic metastases [254]. However, Wnt paracrine signaling between bone stromal cells and metastatic BC cells can also induce osteolytic processes and enhance bone turnover [250]. Furthermore, DKK1 overexpression suppressed lung metastases, promoted osteolytic lesions, and enhanced osteoclast numbers in a murine model of BC. Interestingly, treatment with a canonical Wnt inhibitor enhanced osteolysis and promoted osteoclastogenesis. Investigators saw reduction in both lung and osteolytic metastases when mice were treated with a combination of JNK and TGF-beta inhibitors [255]. These studies indicate that targeting the crosstalk that occurs between multiple cascades likely depends on the balance of signaling that occurs through either canonical or atypical pathways. Successful therapeutic targeting thus requires understanding biomarkers that reflect specific cellular contexts.

### 4.4. Chemokines

PC and BC cells hijack chemokine signaling to invade and metastasize to multiple sites [153,154]. Specifically, the CXCL12-CXCR4 axis normally used by immune cells to home to the hematopoietic bone marrow niche, is appropriated by malignant cells to set up metastatic footholds in the perivascular bone space [153]. Multiple bone stromal cell types secrete chemokines including CCL2, CCL5, and CXCL12 [256,257]. CXCR4 was identified in lung and bone metastasis signatures in BC [258,259] and therefore could potentially modulate invasiveness to other metastatic sites. The CCR6-CCL20 pathway was identified in vertebral BM from patients with PC, and its inhibition prolonged survival in a murine model [127]. The CCL7-CCR3 axis promoted migration of PC cell lines towards human bone marrow-derived adipocytes and, of note, CCR3 expression was enriched in PC metastases to bone as compared to lymph node or visceral metastases [260]. Clinical use of chemokine antagonists will need to account for effects on various immune subsets to avoid inadvertent increase in tumor-promoting immune cells after cessation of therapy. This was demonstrated by Bonapace et al., where co-blockade of IL-6 mitigated unintended effects of CCL2 blockade on immune cells and effectively reduced BC metastases [261].

### 4.5. Other Bone Invasive Pathways

In addition to the pathways described above, multiple other cell adhesion molecules, cytokines, and signaling pathways contribute to BM in solid tumors, and extensive crosstalk occurs between multiple signaling cascades. E-selectin expressed on bone vascular endothelium facilitates attachment of BC cells [154] and induces Wnt signaling [262]. The cell adhesion molecule ALCAM promoted invasion to bone [226]. Osteopontin, a matricellular protein secreted by osteoblasts, interacts with alpha-beta integrins on tumor cells to promote BM, and increased plasma osteopontin correlates with poor prognosis [227,263,264]. Alpha(v)Beta integrin promotes BM in breast [265] and PCs [106]. Connective tissue growth factor (CTGF) interacts with both alpha(v)beta3 integrin and TGF-beta and is associated with BM in prostate and BCs [227,266]. Src has also been implicated in latent BC BM [267,268]. Notch signaling promotes bone lesions in PC [269], BC, and MM [270], and may be targeted by a therapeutic antibody against the Notch ligand Jagged1 [271]. MM cells increase osteocyte autophagic cell death while proteasome inhibitors such as bortezomib (used clinically in MM treatment) improved bone integrity in patients with MM [116]. IL-1beta expressed by PC cells induced cancer-associated-fibroblast related genes in human MSCs, and ablation of IL-1R signaling by an IL-1R antagonist or in IL-1R knockout mice significantly reduced BM in these models [272]. Matrix metalloproteinases are also widely implicated in solid tumor invasion and promote BM and osteoclastogenesis [259,273]. Targeting MMP pathways clinically, however, has thus far yielded minimal benefit (Table 2). Newer approaches leverage the increased expression of proteases and peptidases in tumors, and focus on protease-cleavable active chemotherapeutic agents in antibody-drug-conjugates [274] and on conjugating an MMP inhibitor to a bone-specific bisphosphonate [275]. 

Many of the above pathways signal through phosphoinositide 3-kinase and AKT (protein kinase B) (PI3K/AKT). PI3K is required for differentiation and survival of both osteoblast and osteoclasts [276,277]. In vivo, continuous inhibition of PI3K leads to osteopenia while its deletion impairs postnatal bone acquisition [278]. However, PI3K/AKT promotes progression in multiple cancers, and AKT activation is higher in cells colonizing bone [279,280,281,282,283]. BC cells promoted AKT phosphorylation in osteoclasts, and the PI3K/mTOR-inhibitor PKI-402 inhibited osteoclastogenesis and osteolysis [279]. Activation of AKT in PC BM increased expression of RUNX2 and RUNX2-dependent genes (e.g., MMP9) in tumor cells, facilitating invasion [284]. Furthermore, AKT upregulated RANKL, PTHrP, and BMP2, contributing to crosstalk between PC cells and osteoblasts and osteoclasts [284]. Inhibition of PI3K/AKT in PC prevented BM, downregulated MMP9, and reduced osteolysis [285]. The PI3K inhibitor alpelisib has been approved for patients with metastatic BC harboring PIK3CA-activating mutations [286] and, in one study, about 20% of patients had bone-only metastases and approximately 70% of patients had at least one BM [287]. This suggests that alpelisib could have activity in BM in certain subtypes of cancer, though this remains to be specifically evaluated. 

Extracellular vesicles are important mediators of cell-cell communication by carrying multiple active biomolecules including RNA, DNA, and proteins [288]. Growing evidence supports diverse roles for tumor-derived exosomes in conditioning bone stromal cells to prepare a “premetastatic niche” [289]. Exosomes derived from PC cell lines with different osteolytic abilities and from patients with PC, increased BM burden in a murine model and induced bone stromal cells to secrete the chemokine CXCL12 [290]. Intravenous injection of melanoma-derived exosomes demonstrated accumulation of exosomes in lung and BM and increased metastatic burden at those sites. Furthermore, transplantation of bone marrow cells from mice treated with melanoma-derived exosomes into lethally irradiated mice resulted in significantly greater tumor growth and vascular density of melanoma tumors that were subsequently implanted after bone marrow reconstitution [152]. Several other studies showed that exosomes derived from PC, BC, LC, and MM cells were able to induce osteoblast proliferation and/or osteoclastogenesis and osteoclast proliferation or differentiation [291,292,293,294,295].

### 4.6. Barriers to Discovery of Novel Signaling Pathways in Human Bone Metastatic Cancers

The challenges of obtaining bone biopsies and routine decalcification in post bone biopsy processing have limited discovery of crucial pathways in BM pathophysiology in human cancers. Out of over 500 publicly available datasets deposited in the Gene Expression Omnibus, Sequence Read Archive, and The Cancer Genome Atlas, there were few large-scale BM sequencing projects. Of those datasets and papers that focused on solid tumor cancers, BM secondary to lung and PC dominated, and identified pathways associated with epithelial-mesenchymal transition, immune response, and androgen receptor splice variants [296,297,298,299,300]. These studies employed limited cohorts of between 10–40 patients. The MSK-IMPACT study represents the largest source of genomic data in BM (326 patients with BM, 2 with bone marrow metastases); however, note that this study had 10,000 patients overall [301]. One study in pediatric patients with neuroblastoma examined bone marrow-derived disseminated tumor cells (DTCs) to identify the relapse-seeding clone [302]. Notably, the Human Cancer Metastasis Database (HCMDB) specifically indexes publicly accessible datasets related to metastases transcriptomic data and includes 29 cancer types and 38 sites of metastasis [303]. These data underscore the need for multicellular preclinical models of BM combined with improved bone biospecimen collection protocols to enhance understanding of the complex biological underpinnings of the osseous metastatic niche.

## 5. Future Directions

There are many opportunities to advance interdisciplinary research in understanding and targeting the bone metastatic niche, which span the disciplines of cancer biology, bone biology, and hematology. Much of our understanding of the bone and bone marrow as a tumor environment has emerged from research in malignant bone marrow disorders such as multiple myeloma and leukemia, and understanding the pathways of bone deposition and resorption have already led to treatments that delay and/or prevent SREs. Systemic treatment of solid tumors is increasingly guided by specific genomic alterations within the tumor cells. However, we lack a detailed understanding of how cancer cells with these specific alterations interact with the surrounding stroma. Several preclinical models of bone metastasis highlighted here utilize advantages of 3-D culture systems while continuing to address their limitations in recapitulating the in vivo environment seen in patients. Therefore, biomedical engineering advances will provide an opportunity to complement in vivo models to improve promising hits on preclinical drug screening. Complex and durable ex vivo models provide a promising avenue to study cancer cell invasion and other biologic processes such as tumor dormancy within the bone and bone marrow, using a combination of human primary cells and human cell lines. A combination of ex vivo and in vivo models will remain complementary strategies in the study of BM. Improving preclinical models could lead to discovery of new combination therapies to target BM and identify predictive biomarkers to improve the selection of patients who would most benefit from targeted therapies.

Collaborations between hematology, surgical oncology, medical oncology, radiation oncology, and clinical researchers are crucial to understand clinical manifestations and the treatment of BM, and to obtain patient-derived tissues that are crucial to developing ex vivo human models of bone disease. The use of blanket tissue acquisition IRBs could facilitate collecting human specimens that would normally be discarded (for instance, following hip replacement surgery or orthopedic fixation of traumatic or osteoporotic fractures), and typically have minimal additional risk to the patient or donor. Recently the Boston BM Consortium established an integrated protocol to obtain bone marrow aspirate and tumor tissue from spinal metastases during spinal decompression surgeries for flow cytometry and single cell RNA-sequencing [127]. Collaborative labs could then establish a biobank of primary bone stromal cells from patients with solid tumors, hematologic malignancies, and donors without cancer. Integration of defined workflows that preserve bone biopsies appropriate for genomic and transcriptomic analyses [95,296,297,298,299,300] will be crucial to advance discovery of novel genomic alterations and gene signatures that reveal interactions between tumors and the bone microenvironment. We participated in a multi-institution study to evaluate disseminated tumor cells (DTCs) in bone marrow from patients with localized or metastatic PC, compared to healthy controls [304]. This study demonstrated that DTCs were rare in bone marrow, but illustrated the utility of a multi-institution protocol to obtain primary human bone marrow tissue in patients with cancer. Sourcing primary human bone stromal cells has been accomplished through few avenues. Bone marrow mononuclear cells are commercially available from companies specializing in stem cell culture, however obtaining from multiple donors to assess heterogeneity can be cost prohibitive. At our institution, we have collaborations with the University of Wisconsin Bone Marrow Transplant Program to obtain bone marrow mononuclear cells from healthy donors, performed clinically for bone marrow donation [305]. We have successfully harvested cells remaining in used filters after bone marrow transplantation yielding sufficient cells for multiparametric flow cytometry immunophenotyping [126], live cells to develop bone marrow stromal cultures, and cryobanking. This enables our interdisciplinary group to obtain primary bone stromal and immune cells for immunophenotyping and expansion of ex vivo stromal cultures. Other groups have obtained bone fragments after hip replacement surgeries to successfully expand stromal cells ex vivo. Collaborations with surgical orthopedic oncology would be invaluable in this setting to obtain tissue from fixation of pathologic fractures, as this provides the potential to coculture autologous patient tumor and bone stroma, and to obtain metastatic tissue for multi-omic analysis (genomic, transcriptomic, proteomic).

## 6. Conclusions

Bone metastases are increasing in frequency, in part due to improved imaging modalities and to improved survival, as systemic therapies improve in multiple solid tumor types. Given that bone metastases decrease quality of life and are associated with poor survival in several malignancies, new therapies are urgently needed. Remaining questions include optimal timing to initiate antimetastatic therapy (adjuvant, early vs. late metastatic disease), duration of therapy, safety and efficacy of combining antimetastatic therapy with concurrent tumor-specific standard treatments (such as hormone therapy, targeted therapy, chemotherapy, and immunotherapy, radiotherapy) and appropriate clinical trial endpoints. 

Improved preclinical models of the complex bone environment are urgently needed to develop effective therapies. Microfluidic organ-on-chip platforms that enable ex vivo culture and treatment of patient-derived cancer and stromal cells have strong potential to bridge the gap between 2-D coculture approaches and in vivo mouse models. Advancing microscale techniques has enabled investigators to better recapitulate complex multicellular stroma composition and mimic microvasculature and 3-D organoid biology. To enhance the clinical relevance of microscale preclinical models, primary human tissues will need to be obtained, which depends on increasing collaboration between multiple disciplines. Given the poor prognosis and significant impact of bone metastases on morbidity and mortality, understanding the bone metastatic niche is an area of research ripe for discovery with clear clinical relevance and potential to greatly improve clinical outcomes.
